# VEPTP inhibition with an extracellular domain targeting antibody did not restore albuminuria in a mouse model of diabetic kidney disease

**DOI:** 10.14814/phy2.70058

**Published:** 2024-09-26

**Authors:** Rajashree Rana, Thomas A. Natoli, Puneet Khandelwal, Pavlos Pissios, Abdul Bari Muhammad, Vaja Chipashvili, Krista P. Farrington, Wen Zhou, Gang Zheng, Nikolay O. Bukanov, Alessandro Pocai, Maria Chiara Magnone

**Affiliations:** ^1^ Cardiovascular and Metabolism, Johnson & Johnson Research & Development Spring House Pennsylvania USA; ^2^ Cardiovascular and Metabolism, Johnson & Johnson Research & Development Cambridge Massachusetts USA; ^3^ Biologics Discovery, Johnson & Johnson Research & Development Spring House Pennsylvania USA

**Keywords:** diabetic kidney disease, protein tyrosine phosphatase receptor type B, renal impairment, vascular endothelial protein tyrosine phosphatase

## Abstract

Diabetic kidney disease (DKD) is the leading cause of end‐stage kidney disease. DKD is a heterogeneous disease with complex pathophysiology where early endothelial dysfunction is associated with disease progression. The Tie2 receptor and Angiopoietin 1 and 2 ligands are critical for maintaining endothelial cell permeability and integrity. Tie2 signaling is negatively regulated by the endothelial specific transmembrane receptor Vascular Endothelial Protein Tyrosine Phosphatase (VEPTP). Genetic deletion of VEPTP protects from hypertension and diabetes induced renal injury in a mouse model of DKD. Here, we show that VEPTP inhibition with an extracellular domain targeting VEPTP antibody induced Tie2 phosphorylation and improved VEGF‐A induced vascular permeability both in vitro and in vivo. Treatment with the VEPTP blocking antibody decreased the renal expression of endothelial activation markers (*Angpt2*, *Edn1*, *and Icam1*) but failed to improve kidney function in db/db uninephrectomized ReninAAV DKD mice.

## 1 INTRODUCTION

Diabetic kidney disease (DKD) is the leading cause of end‐stage renal disease (ESRD) worldwide (Afkarian et al., 2016; Forst et al., 2022; Fu et al., 2019; Li et al., 2021; Oshima et al., 2021), representing an enormous economic burden. DKD is characterized by albuminuria and an initial phase of glomerular hyperfiltration followed by progressive decline of glomerular filtration rate (GFR). Pathogenesis of DKD is multifactorial and involves numerous metabolic, inflammatory, and hemodynamic pathophysiological processes. One of the earliest events during chronic hyperglycemia is endothelial dysfunction, leading to albuminuria, changes in GFR (Satchell, 2012; Toyoda et al., 2007; Weil et al., 2012) and progression of DKD (Clausen et al., 1999; Stehouwer et al., 1995). In Pima Indians with T2DM, reduction in glomerular endothelial fenestrae correlated with the level of albuminuria and loss of GFR (Weil et al., 2012). The current standard‐of‐care treatment for DKD include the use of angiotensin‐converting enzyme (ACE) inhibitors, angiotensin receptor blockers (ARBs) and potentially sodium‐glucose cotransporter‐2 (SGLT‐2) inhibitors that primarily regulate hemodynamics and hyperglycemia (Brenner et al., 2001; Duckworth et al., 2009; Heerspink et al., 2020; Lewis et al., 2001; Muskiet et al., 2019; Perkovic et al., 2019; Sen & Heerspink, 2021; Wanner et al., 2016). Although, the use of these drugs slow kidney function decline, many patients continue to progress to ESRD. Progression of microvascular complications in DKD is complex and emerging evidence support an important role of endothelium‐specific Tie2 (tyrosine kinase with IgG and EGF homology domains 2) receptor, Angiopoietin‐1 (*ANGPT1* or Ang‐1) and Angiopoietin‐2 (*ANGPT2* or Ang‐2) ligands. Ang‐1 functions as an agonistic ligand of Tie2 and its loss results in glomerular damage in diabetic mice (Jeansson et al., 2011). It has been shown that adenoviral delivery of cartilage oligomeric matrix protein‐Ang1 (COMP‐Ang1) decreased albuminuria and delayed fibrotic changes in the kidneys of diabetic mice (Lee et al., 2007). Conversely, Ang‐2 functions as a context dependent antagonistic ligand. It has been reported that plasma concentration of Ang‐2 is higher in patients with diabetes and correlates positively with urinary albumin: creatinine ratio (uACR) (Chang et al., 2013; Lim et al., 2005). Furthermore, *ANGPT2* mRNA was increased in the glomeruli of patients with diabetic nephropathy (Dessapt‐Baradez et al., 2014; Shi et al., 2018) and a decrease in Ang‐1/Ang‐2 ratio contributed to disease progression in preclinical animal models of DKD (Rizkalla et al., 2005; Yamamoto et al., 2004).

In addition to the angiopoietin ligands, Tie2 activity is regulated by the endothelial specific transmembrane receptor‐type protein tyrosine phosphatase Vascular Endothelial Protein Tyrosine Phosphatase (VEPTP), which potently dephosphorylates Tie2 and functions as a negative regulator of Tie2 mediated cell signaling (Bäumer et al., 2006; Dominguez et al., 2007; Fachinger et al., 1999; Krueger et al., 1990; Vestweber, 2021). The VEPTP receptor consists of an extracellular fibronectin type III‐like (FN‐III) repeats enriched domain, a transmembrane and an intracellular phosphatase domain. The major substrates of VEPTP include Tie2 and the endothelial specific adhesion molecule VE‐cadherin (Fachinger et al., 1999; Frye et al., 2015; Nawroth et al., 2002; Saharinen et al., 2008). VEPTP associates with Tie2 through cytoplasmic interactions (Fachinger et al., 1999; Saharinen et al., 2008), whereas VEPTP and VE‐cadherin interact via their membrane‐proximal extracellular domains (ECD) (Nawroth et al., 2002).

Both monoclonal and polyclonal VEPTP antibodies generated by immunization with the N‐terminal protein fragment antigens have been described (Bäumer et al., 2006; Winderlich et al., 2009). These antibodies are large, cell impermeable molecules and cannot access the intracellular VEPTP phosphatase domain. They bind the ECD, likely causing conformational changes which in turn regulate receptor phosphatase activity and downstream signaling. Anti‐VEPTP antibodies were demonstrated to mimic the effects of *VEPTP* gene disruption resulting in Tie2‐activation and vessel enlargement in allantois explants (Bäumer et al., 2006; Senis & Barr, 2018; Winderlich et al., 2009). VEPTP small molecule inhibitor AKB‐9778 was tested in clinic for diabetic macular edema and in the trial a 20% reduction in uACR was observed (Campochiaro et al., 2015; Campochiaro et al., 2016; Kevin et al., 2018).

In addition to pharmacological inhibitors of VEPTP, it has been shown that genetic deletion of VEPTP restores Tie2 activity and decreases albuminuria in the Akita‐Renin DKD mice (Carota et al., 2019). Here, we explore the impact of VEPTP inhibition using an ECD‐targeting VEPTP antibody in the db/db uninephrectomized (Unx) ReninAAV mouse model of DKD.

## 2 MATERIALS AND METHODS

### 2.1 Study drug

The hybridoma cell line producing antibodies against mouse VEPTP, with the clone number 109.1 was obtained from the lab of Professor Dr. Dietmar Vestweber, Max‐Planck Innovation GmbH (Germany). The rat hybridoma cell line 109.1 producing a rat IgG2a against mouse VEPTP recognizes an epitope within the N‐terminal 8 FNIII‐like domains of VEPTP and was generated against a VEPTP‐Fc construct (Bäumer et al., 2006). The subclone 109.3 (different subclone from the same mother clone) and 109.1 have been described previously (Bäumer et al., 2006; Winderlich et al., 2009). The VEPTP antibody 109.1 was produced from the hybridoma cells, purified, and tested for endotoxins and pathogens (Charles River) before administration to mice.

### 2.2 Cell culture and phospho‐Tie2 (Tyr 992) and phospho‐ERK1/2 (Thr 202/Tyr204) measurements

Mouse endothelial cells bEnd.3 (ATCC, CRL‐2299) were cultured in Dulbecco's modified Eagle's medium (DMEM, Gibco, 11995065) supplemented with 10% FBS (Gibco, 10082147) and 1% penicillin/streptomycin (Gibco, 15140148) in a humidified environment with 5% CO_2_ at 37°C. For treatment with the VEPTP blocking antibody mAb 109.1, bEnd.3 cells were seeded in serum containing DMEM and allowed to grow overnight. Next morning the bEnd.3 cells were serum starved for 4 h and then treated with different concentrations of the mAb 109.1 or isotype control (ThermoFisher Scientific, PA5‐33214) for 30 min in serum free DMEM containing 0.5% of BSA. After treatment completion, cells were lysed, and lysate collected for downstream phospho‐protein analyses.

Phospho and total Tie2 were measured using Meso Scale Discovery (MSD)‐based assays, the capture antibodies used were phospho‐Tie2 (PA5‐104669, ThermoFisher Scientific) and total Tie2 (R&D Systems, BAF762). The detection antibody used was 14‐5987‐82, ThermoFisher Scientific. The phospho‐Tie2 antibody was biotinylated using a biotinylation kit (ThermoFisher Scientific, 90407). Phospho‐ERK1/2 (Revvity, ALSU‐PERK‐A500) and total ERK1/2 (Revvity, ALSU‐TERK‐A500) were measured using AlphaLISA based methods following manufacturer's instructions.

### 2.3 In vitro vascular permeability assay

To determine paracellular permeability, 2 × 10^4^ mouse endothelial cells (bEnd.3, ATCC CRL‐2299) were seeded on the coated inserts (Millipore Sigma, ECM644) in complete DMEM media and grown to confluence for 72 h at 37°C and 5% CO_2_. The inserts contain 1 μm pores within a transparent polyethylene terephthalate (PET) membrane. Each insert has been pre‐coated with an optimized concentration of type I rat‐tail collagen. For treatment with the VEPTP blocking antibody mAb 109.1, bEnd.3 cells were serum starved for 2 h and treated with mAb 109.1 (50 nm and 100 nM) in the presence or absence of 500 ng/mL mouse VEGF‐A (R&D Systems, 493‐MV) for 22 h. After 22 h of treatment, media containing mAb 109.1 and VEGF‐A was removed and DMEM containing a high molecular weight FITC‐dextran was added to the upper chamber of the inserts and DMEM without FITC‐dextran was added to the lower chamber. After 30 min of FITC‐dextran treatment, media from the lower chamber was collected and fluorescence measured using a SpectraMax Plate reader. Vascular permeability was calculated as percent change from baseline (fluorescence signal in media without VEGF‐A and/or antibody treatment).

### 2.4 In vitro gene analyses (quantitative real‐time PCR)

Mouse endothelial cells (bEnd.3) were seeded in serum containing DMEM and allowed to grow overnight. Next morning, the bEnd.3 cells were serum starved for 2 h and then treated with the VEPTP blocking antibody mAb 109.1 (50 nm and 100 nM) for 24 h in serum free DMEM. After treatment completion, cells were lysed in Cells‐to‐CT‐bulk lysis reagents (ThermoFisher Scientific, 4391851C) and lysate collected for gene expression analyses using RNA‐to‐CT 1‐step Kit (ThermoFisher Scientific, 4392938). The expression of the genes of interest *Angiopoietin 2* (*Angpt2*) and *Endothelin 1* (*Edn1*) were calculated relative to the expression of the house keeping gene *Peptidylprolyl isomerase A* (*Ppia*). The Taqman probes used and their respective catalogue numbers are summarized in Table 1. ΔΔCT data were represented as relative‐fold changes in mRNA normalized to the house‐keeping gene.

**TABLE 1:** Taqman gene expression assay probes.

Gene symbol	Gene name	Assay ID (catalogue number)
*Angpt2*	*Angiopoietin 2*	Mm00545822_m1
*Edn1*	*Endothelin 1*	Mm00438656_m1
*Icam1*	*Intercellular adhesion molecule 1*	Mm00516023_m1
*Vcam1*	*Vascular cell adhesion molecule 1*	Mm01320970_m1
*Igfbp7*	*Insulin‐like growth factor binding protein 7*	Mm03807886_m1
*Fn1*	*Fibronectin 1*	Mm01256744_m1
*Ctgf*	*Connective tissue growth factor*	Mm01192933_g1
*Acta2*	*Smooth muscle Actin*	Mm00725412_s1
*Col1a1*	*Collagen type 1 alpha 1*	Mm00801666_g1
*Ccl2*	*Chemokine* (*C‐C motif*) *ligand 2*	Mm00441242_m1
*Lcn2*	*Lipocalin 2*	Mm01324470_m1
*Havcr1*	*Hepatitis A virus cellular receptor 1*	Mm00506686_m1
*Ppia*	*Peptidylprolyl isomerase A*	Mm02342430_g1

### 2.5 Animals

All animal experiments were performed in accordance with the Institutional Animal Care and Use Committee (IACUC) and Charles River Accelerator and Development Lab (CRADL) guidelines. C57BL/6J female mice (7–10‐week‐old) were purchased from Jackson Laboratories and used in the in vivo acute dermal vascular permeability and pharmacokinetic studies as described below. Uninephrectomized female db/db mice (7–8‐week‐old) were also purchased from Jackson Laboratories (BKS.Cg‐Dock7m +/+ Leprdb/J; strain# 000642) and aged to 11–12‐weeks to facilitate disease progression before viral injections as described below. All mice were housed in temperature‐controlled rooms (22°C) with 12/12‐h light/dark cycle. Mice had ad libitum access to standard chow (LabDiet, 5008) and tap water. After the completion of all in vivo experiments, mice were euthanized by exsanguination under deep anesthesia, followed by cervical dislocation.

### 2.6 In vivo dermal vascular permeability assay

C57BL/6J female mice were treated with mAb 109.1 (100 μg) or isotype (ThermoFisher Scientific, PA5‐33214) via retroorbital injections 30 min prior to Evans Blue dye (Sigma, E2129) administration. 100 μL of the Evans Blue dye (1% weight/volume) solution was administered retro‐orbitally following isoflurane inhalation. After 10 min of Evans Blue administration, acute, local dermal vascular hyperpermeability was induced by 100 ng transdermal VEGF‐A (R&D Systems, 493‐MV) injection. Thirty minutes after VEGF‐A administration, mice were euthanized, and skin tissue excised. Lung tissues were collected, and flash frozen for phospho‐protein analyses. Skin biopsies were dried for 24 h at 55°C. To extract the Evans Blue dye from the skin samples, de‐ionized formamide (Sigma‐Aldrich, 221198) was added and samples incubated at 55°C for 24 h. Formamide containing Evans Blue dye exuded from skin biopsies were quantified by measuring absorbance (620 nm) using a SpectraMax Plate reader.

### 2.7 Phospho‐Tie2 (Tyr 992), phospho‐AKT1/2/3 (ser 473) and phospho‐ERK1/2 (Thr 202/Tyr 204) measurements in tissue samples

Approximately 20–25 mg of lung or kidney tissues were lysed and homogenized in lysis buffer containing protease and phosphatase inhibitors (ThermoFisher Scientific, 78442). Phospho and total Tie2 were measured using MSD based methods as described above. Phospho‐AKT 1/2/3 (Revvity, ALSU‐PAKT‐B500), total AKT1/2/3 (Revvity, ALSU‐TAKT‐B500), phospho‐ERK1/2 (Revvity, ALSU‐PERK‐A500) and total ERK1/2 (Revvity, ALSU‐TERK‐A500) were measured using AlphaLISA based methods following manufacturer's instructions.

### 2.8 Single and multiple dose pharmacokinetic studies in C57BL/6J female mice

For the single dose pharmacokinetic (PK) and target engagement experiments, C57BL/6J (7–8‐week‐old) female mice were injected subcutaneously with a single dose of vehicle (PBS) or mAb 109.1 at three different doses (5, 10, and 20 mg/kg). Following mAb 109.1 administration, mice were euthanized either on Days 1, 2, 4, 7 or 14. Whole blood was collected by cardiac puncture and separated for plasma. Plasma concentrations of mAb 109.1 were measured by ELISA (Abcam, ab157737) based methods. Kidney tissues were collected, flash frozen and stored at −80°C for target engagement analyses. Phospho‐Tie2 and total Tie2 in kidney tissues were measured using MSD based methods as described above. Kidney gene expression analyses was performed as described below.

For the multiple dose PK experiments, C57BL/6J (8–10‐week‐old) female mice were injected subcutaneously with two different doses of mAb 109.1 (5 and 20 mg/kg) twice/week for 4‐weeks. Plasma was collected on Days 2, 15 and 29 by submandibular bleeds and plasma concentrations of the mAb 109.1 measured using ELISA based methods.

### 2.9 Mouse model of diabetic nephropathy and drug treatments

The in vivo *proof‐of‐concept* experiments with mAb 109.1 and standard of care lisinopril (BioVision, B2132‐250) were performed in the female db/db uninephrectomized ReninAAV mouse model of DKD. Female db/db mice were used to reduce the risk of hyperglycemia induced pyelonephritis. The male diabetic mice are more prone to hyperglycemia induced pyelonephritis because of anatomical differences rendering male mice more prone compared to female mice. An AAV construct, AAV8‐TBG‐m‐Ren1d(F61R/P65S) (Vector Biolabs), was used to induce hypertension in female db/db mice following uninephrectomy (Unx). ReninAAV construct was suspended in sterile PBS and a single dose (1e^10^) was administered retro‐orbitally while under isoflurane anesthesia. Ninety‐nine animals were injected with virus to account for attrition (~25%) and to meet inclusion criteria. Treatment was initiated 4 weeks after ReninAAV administration. Animals were randomized based on baseline uACR, systolic blood pressure (SBP), blood glucose (BG) and body weight (BW) before the treatment start. Inclusion criteria: uACR ≥3000 μg/mg, BG ≥250 mg/dL, SBP ≥110 mmHg, and BW ≥38 g. Randomization was performed by assigning the following weightage to the baseline measurements uACR (0.7), SBP (0.1) BG (0.1), and BW (0.1). db/db Unx ReninAAV mice (*n* = 16 mice/group) received either subcutaneous (s.c.) vehicle (PBS), s.c. vehicle (PBS) + P.O. lisinopril (10 mg/kg) in drinking water, or s.c. mAb 109.1 at two different doses (5 mg/kg or 20 mg/kg) for 4 weeks, dosing twice/week. Mice were euthanized after 4 weeks of treatment corresponding to Week 8 after ReninAAV administration.

Body weight was measured weekly and water consumption was measured twice/week for the lisinopril treatment group to measure lisinopril dose. VEPTP doses were selected from the single dose pharmacokinetics experiments and lisinopril dose was within standard ranges previously used for mouse models of DKD (Wu et al., 2022). After 4 weeks of treatment, animals were euthanized by exsanguination under deep anesthesia, followed by cervical dislocation. Whole blood was collected by cardiac puncture and separated for plasma for measurements of mAb 109.1, creatinine and blood urea nitrogen (BUN). Urine samples were collected using metabolic cages and used for uACR measurements. Kidney samples were collected flash frozen and stored at −80°C for gene expression analyses as described below.

### 2.10 Blood and urine analyses

Blood glucose was measured using a LifeScan OneTouch Ultra glucometer according to the manufacturer's instructions. Plasma creatinine and urea were measured using LC–MS (Sciex, QTrap 5500) based methods. Urine creatinine and albumin were measured using the Alfa Wasserman Chemistry Analyzer. mAb 109.1 plasma exposure was measured by ELISA (Abcam, ab157737) based methods.

### 2.11 RNA isolation from tissues and quantitative PCR

Total RNA was isolated from mouse kidneys using the RNeasy Plus Mini Kit (Qiagen, 74136) following manufacturer's instructions. Real time quantitative PCR (qPCR) of genes of interest was conducted using the Taqman RNA‐to‐Ct 1‐Step Kit (ThermoFisher Scientific, 4392938) on a QuantStudio 12 or 7 Flex Real‐Time PCR System (Applied Biosystems) and a 40‐thermocycling protocol. ΔΔCT data were represented as relative fold changes in mRNA normalized to the house‐keeping gene *Peptidylprolyl isomerase A* (*Ppia*). The catalogue numbers of the Taqman gene expression assay probes are as summarized in Table 1. All probes were obtained from ThermoFisher Scientific.

### 2.12 Statistics

Statistical analyses were performed using GraphPad prism v9.0 (GraphPad). All results are shown as mean ± SEM. Data were analyzed by Ordinary one‐way ANOVA followed by Dunnett's multiple comparisons test. *p* ≤ 0.05 was considered statistically significant.

## 3 RESULTS

### 3.1 VEPTP inhibition with an extracellular domain targeting antibody mAb 109.1 induces Tie2 and ERK1/2 phosphorylation and protects against VEGF‐A induced vascular permeability in mouse endothelial cells

**FIGURE 1:**
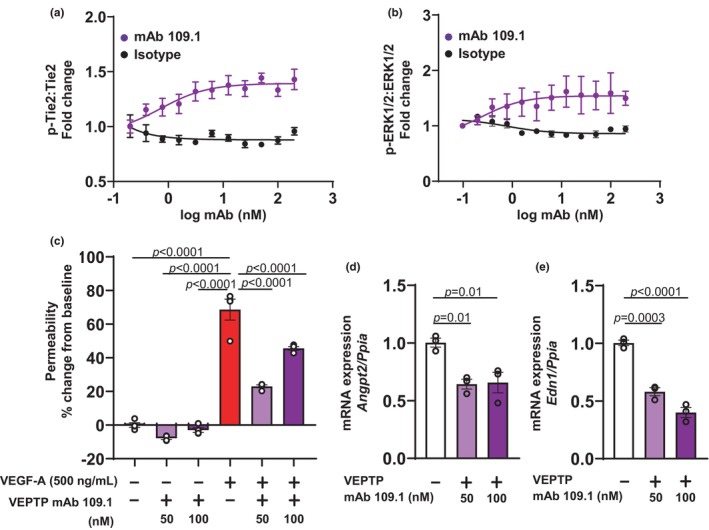
VEPTP inhibition with an extracellular domain targeting antibody mAb 109.1 induces Tie2 and ERK1/2 phosphorylation and protects against VEGF‐A induced vascular permeability in mouse endothelial cells. (a) Mouse endothelial cells (bEnd.3, ATCC) were treated with increasing concentrations of mAb 109.1 (purple circles) or the isotype (black circles). mAb 109.1 treatment resulted in a dose dependent increase in phospho‐Tie2:total Tie2 ratio. (b) mAb 109.1 treatment (purple circles) resulted in a dose‐dependent increase in the phospho‐ERK1/2:ERK1/2 ratio. (c) VEGF‐A (500 ng/mL) treatment significantly increased permeability of FITC‐dextran across confluent mouse endothelial monolayer on transwell membranes. VEGF‐A induced endothelial monolayer permeability was significantly blocked by mAb 109.1 treatment at both 50 and 100 nM concentrations. (d, e) qPCR analyses showed that mAb 109.1 treatment (at 50 and 100 nM) of mouse endothelial cells resulted in a significant decrease in *Angpt2* and *Edn1* expression. VEPTP, vascular endothelial protein tyrosine phosphatase; Tie2, tyrosine kinase with immunoglobulin and epidermal growth factor homology domains 2; ERK1/2, extracellular signal‐regulated kinase 1/2; VEGF‐A, vascular endothelial growth factor A; FITC‐dextran, fluorescein isothiocyanate dextran; qPCR, quantitative polymerase chain reaction; *Angpt2*, *angiopoietin‐2*; *Edn1*, *endothelin‐1*; *Ppia*, *Peptidylprolyl isomerase A*.

The VEPTP extracellular domain targeting monoclonal antibody mAb 109.1 recognizes an epitope within the N‐terminal 8 FNIII‐like domains of VEPTP (Bäumer et al., 2006; Winderlich et al., 2009). Here, we determined the potency of the mAb 109.1 in endothelial cells by measuring the phosphorylation of Tie2 (Tyr992). Treatment of mouse endothelial cells (bEnd.3) with increasing concentrations of mAb 109.1 resulted in a dose‐dependent increase in phospho‐Tie2:total Tie2 ratio with an EC_50_ of 0.7 nM (Figure 1a). No induction of Tie2‐phosphorylation was observed upon treatment with the isotype control. As shown in Figure 1b, a dose dependent increase in phospho‐ERK1/2:ERK1/2 ratio was noted with mAb 109.1 but not with control isotype. Phospho‐Tie2, total Tie2 and phospho‐ERK1/2, total ERK1/2 were measured using MSD and AlphaLISA based methods, respectively.

Next, we investigated the effect of VEPTP inhibition on VEGF‐A induced endothelial cell permeability. Mouse endothelial cells (bEnd.3) were grown to confluence on permeable transwell inserts for 72 h resulting in the formation of an endothelial monolayer with tight junctions. As shown in Figure 1c, addition of VEGF‐A (500 ng/mL) to the endothelial monolayer disrupted the tight junctions and induced cell permeability as measured by the increased permeability of FITC‐dextran to the adjacent lower chamber resulting in a significant 68.6% increase in fluorescent signal compared to no VEGF‐A treatment. The VEPTP blocking mAb 109.1 at both 50 and 100 nM concentrations provided significant reduction in VEGF‐A induced endothelial monolayer permeability (Figure 1c).

Because VEPTP inducible knockout mice exhibited decreased expression of FOXO1 target genes *Angiopoietin 2* (*Angpt2*) and endothelin‐1 (*Edn1*) (Carota et al., 2019), we evaluated whether inhibition of VEPTP with mAb 109.1 manifested similar effects on *Angpt2* and *Edn1*. Mouse endothelial cells were grown to confluence, serum starved for 2 h and treated with the mAb 109.1 (50 nm and 100 nM) for 24 h. As shown in Figure 1d,e, mAb 109.1 treatment at 50 and 100 nM doses resulted in a significant 35%–36% decrease in *Angpt2* mRNA and 42%–60% decrease in *Edn1* mRNA (*p* ≤ 0.0001).

### 3.2 VEPTP inhibition with mAb 109.1 provided protection against VEGF‐A induced dermal vascular leakage in mice and induced Tie2‐phosphorylation in lung

**FIGURE 2:**
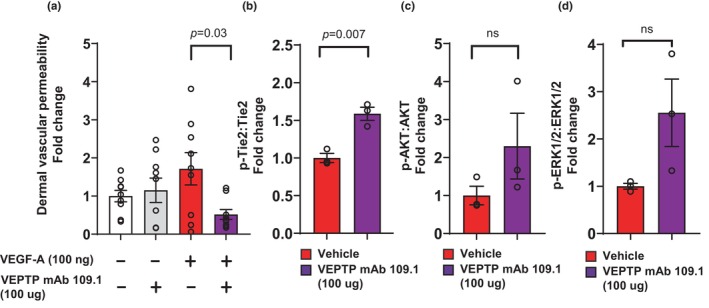
VEPTP inhibition with mAb 109.1 provided protection against VEGF‐A induced dermal vascular leakage in C57BL/6J female mice and induced Tie2‐phosphorylation in lung. (a) C57BL/6J female mice were injected s.c. with mAb 109.1 (100 μg) or isotype 30 min prior to Evans Blue administration. VEGF‐A induced Evans blue leakage was significantly decreased with mAb 109.1 pre‐treatment. (b) mAb 109.1 pre‐treatment resulted in a significant increase in phospho‐Tie2:total Tie2 ratio. mAb 109.1 pre‐treatment resulted in non‐significant trends in increase in (c) phospho‐AKT1/2/3 (Ser473)/AKT1/2/3 and (d) phospho‐ERK1/2 (Thr202/Tyr204)/ERK1/2 ratio. *n* = 3 mice per treatment group. VEPTP, vascular endothelial protein tyrosine phosphatase; VEGF‐A, vascular endothelial growth factor A; s.c., subcutaneous; Tie2, tyrosine kinase with immunoglobulin and epidermal growth factor homology domains 2; AKT1/2/3, akt serine threonine kinase 1/2/3; ERK1/2, extracellular signal‐regulated kinase 1/2.

Because VEPTP inhibition with mAb 109.1 provided protection against VEGF‐A induced vascular permeability in vitro, we tested the effects of VEPTP inhibition in vivo. It has been reported that VEPTP blockade with a small molecule targeting the intracellular phosphatase domain of the receptor suppresses VEGF‐A and histamine induced vascular leakage (Shen et al., 2014). Here, we tested whether targeting the extracellular domain of VEPTP with a monoclonal antibody also elicited similar effects in providing protection against VEGF‐A induced dermal vascular permeability. The Miles assay was used to test vascular permeability in vivo. C57BL/6J female mice were administered 100 μg of the VEPTP antibody intravenously (*i.v*.) via retro‐orbital injections 30 min prior to start of the assay. Evan's blue dye was injected to the mice *i.v*., and 10 min later was followed by transdermal injections of mouse recombinant VEGF‐A. Thirty minutes after VEGF‐A injection mice were euthanized, Evan's blue dye extracted from the skin biopsies and quantified. Indeed, as shown in Figure 2a, VEGF‐A induced dermal vascular leakage in mice was significantly reduced by pre‐treatment with the VEPTP blocking mAb 109.1.

Lung tissues were collected from the mice injected with vehicle or VEPTP antibody and analyzed for phosphorylation of Tie2 (Tyr 992), AKT1/2/3 (Ser 473), and ERK1/2 (Thr202/Tyr204). As shown in Figure 2b, VEPTP inhibition with mAb 109.1 resulted in a significant increase in the phospho‐Tie2:total Tie2 ratio. As shown in Figure 2c,d, VEPTP inhibition with mAb 109.1 induced phosphorylation of AKT1/2/3 and ERK1/2 in lungs.

### 3.3 Systemic pharmacokinetics, kidney Tie2 phosphorylation and gene expression following mAb 109.1 administration in C57BL/6J female mice

**FIGURE 3:**
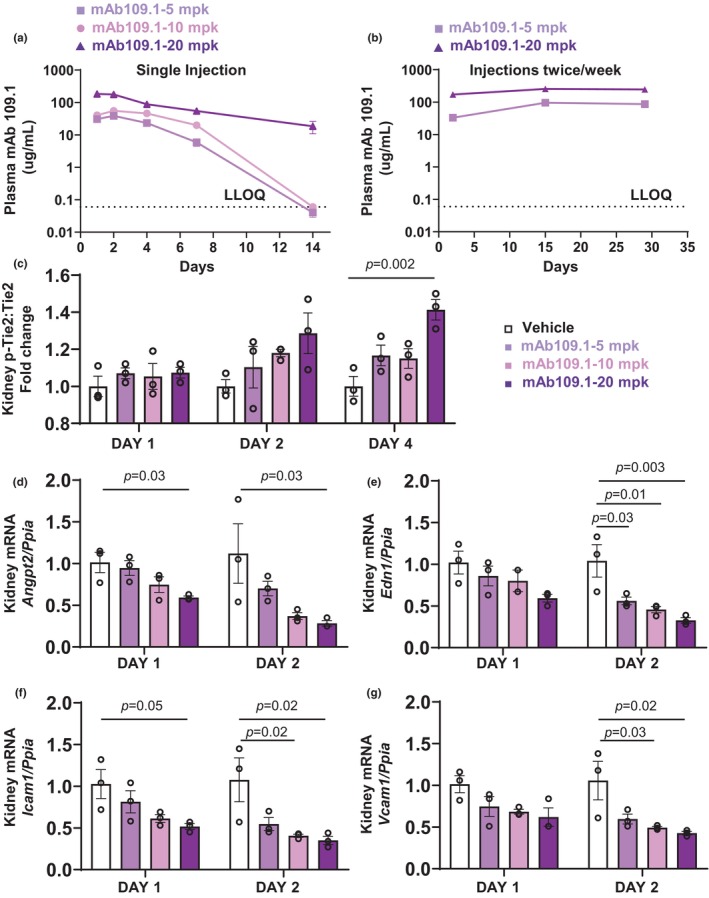
Systemic pharmacokinetics, kidney Tie2 phosphorylation and gene expression following mAb 109.1 administration in C57BL/6J female mice. (a) Plasma concentration‐time profile of mAb 109.1 after single dose s.c. administration of 5, 10 and 20 mg/kg antibody measured on Days 1, 2, 4, 7 and 14 using ELISA‐based methods. The mAb 109.1 was detected in plasma for 1–4 days at all three‐doses. After Day 4, decline in plasma concentrations of the antibody were noted and at Day 14, mAb 109.1 concentrations were below lower limit of quantification (LLOQ) for the two lower doses (5 and 10 mg/kg). (b) Plasma concentration‐time profile of mAb 109.1 after multiple dose administration of the antibody. C57BL/6J female mice were injected s.c. twice per week for 4‐weeks with two doses (5 and 20 mg/kg) of the mAb 109.1. Plasma mAb 109.1 concentrations were measured on Days 2, 15 and 29 following antibody administration. Systemic concentrations of the antibody were maintained for 4‐weeks following repeated dose s.c. administration of the antibody in mice. (c) Kidneys were collected from the mice injected with the single dose of the mAb 109.1 and analyzed for phospho‐Tie2 (Tyr992) and Tie2. mAb 109.1 treatment at 20 mg/kg resulted in a significant increase in phospho‐Tie2:total Tie2 ratio on Day 4. (d–g) Kidneys were collected from the mice injected with single dose of mAb 109.1 and analyzed for *angiopoietin‐2* (*Angpt2*), *endothelin 1* (*Edn1*), *intercellular adhesion molecule 1* (*Icam1*) and *vascular cell adhesion molecule 1* (*Vcam1*) mRNA expression by qPCR. A dose‐dependent decrease in *Angpt2*, *Edn1*, *Icam1 and Vcam1* expression were noted with mAb 109.1 treatment. *n* = 3–5 mice per treatment group. Tie2, tyrosine kinase with immunoglobulin and epidermal growth factor homology domains 2; s.c., subcutaneous; AKT1/2/3, akt serine threonine kinase 1/2/3; ERK1/2, extracellular signal‐regulated kinase 1/2; *Ppia*, *Peptidylprolyl isomerase A*.

We determined the systemic pharmacokinetic profile of mAb 109.1 following single and multiple dose *s.c*. administration. The plasma concentration‐time profiles for the different doses of the antibody following a single dose administration are as shown in Figure 3a, there was a direct correlation between the mAb 109.1 dose administered and plasma concentrations; wherein the highest dose administered (20 mg/kg) resulted in highest plasma concentrations of mAb 109.1 and the lowest dose (5 mg/kg) exhibited lowest plasma concentrations of the antibody. After Day 4, decline in plasma concentrations of mAb 109.1 was noted and between Days 7 and 15, mAb 109.1 was eliminated from plasma for the two lower doses (5 and 10 mg/kg) of the antibody administration exhibiting non‐linear pharmacokinetics.

Next, we determined systemic exposures of mAb 109.1 following multiple dose administration. C57BL/6J female mice were injected twice per week for 4 weeks with two doses of the mAb 109.1 (5 and 20 mg/kg), plasma samples were collected on Days 2, 15 and 29 and VEPTP blocking mAb 109.1 concentrations determined as described earlier. As shown in the plasma concentration versus time profiles in Figure 3b, the plasma concentrations of the antibody were maintained for 4 weeks following repeated dose administration in mice. As shown in Figure 3c, single dose *s.c*. administration of mAb 109.1 induced kidney Tie2‐phosphorylation.

We further determined the gene expression of endothelial activation markers (*Angpt2*, *Edn1*, *Icam1* and *Vcam1*) in the kidneys of mice injected with a single dose of the anti‐VEPTP antibody on Days 1 and 2 as proximal pharmacodynamic kidney biomarkers. Indeed, as shown in Figure 3d–g, a dose dependent decrease in kidney *Angpt2*, *Edn1*, *Icam1 and Vcam1* gene expression were noted with the VEPTP blocking mAb 109.1 treatment on both Days 1 and 2. The maximum effect was noted on Day 2, with a 72%, 67%, 65% and 57% decrease in the expression of *Angpt2*, *Edn1*, *Icam1 and Vcam1* respectively with the VEPTP mAb 109.1 treatment at 20 mg/kg.

### 3.4 Effects of VEPTP inhibition with mAb 109.1 in the db/db uninephrectomized ReninAAV mice model for DKD

It has been shown previously that genetic deletion of VEPTP (*Ptprb*) in mouse provided protection against acute and chronic renal injury (Carota et al., 2019; Li et al., 2023). In the current study, we tested whether pharmacological inhibition of VEPTP with mAb 109.1 resulted in renal function improvement in the db/db Unx ReninAAV mouse model of severe DKD. Severe progressive DKD was established by adeno‐associated virus (AAV) mediated hepatic overexpression of renin in uninephrectomized (Unx) homozygous *lepr* knockout (db/db) female mice. The db/db Unx ReninAAV mice manifests severe renal dysfunction characterized by increased albuminuria, increased serum creatinine and BUN (Harlan et al., 2015; Harlan, Heinz‐Taheny, Overstreet, et al., 2018; Harlan, Heinz‐Taheny, Sullivan, et al., 2018; Østergaard et al., 2021; Wu et al., 2022).

**FIGURE 4:**
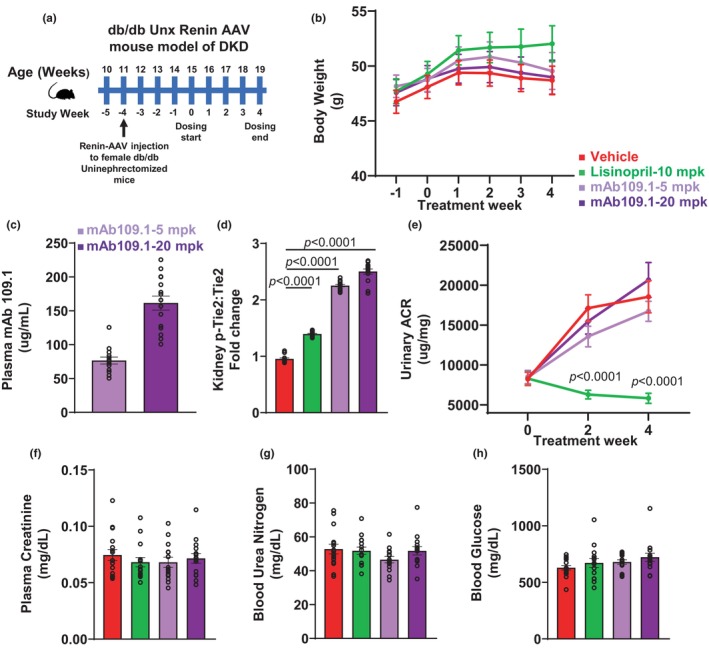
Effects of VEPTP inhibition with mAb 109.1 in the db/db uninephrectomized ReninAAV mice model for DKD. (a) Experimental scheme depicting the timeline and age of mice corresponding to Renin‐AAV injection and dosing with mAb 109.1 or lisinopril. Dosing with mAb 109.1, vehicle or lisinopril was initiated 4 weeks after Renin‐AAV injection and continued for 4‐weeks. (b) Body weight monitoring of the db/db uninephrectomized ReninAAV mice over the course of dosing with mAb 109.1 or lisinopril. (c) db/db Unx ReninAAV female mice were treated with two doses (5 and 20 mg/kg) of mAb 109.1, administered s.c., twice weekly for 4‐weeks. At the end of the study, plasma concentrations of mAb 109.1 were measured using ELISA‐based methods. (d) After treatment completion, kidneys were analyzed for phospho‐Tie2 (Tyr992) and total Tie2. Lisinopril and mAb 109.1 treatment at both 5 and 20 mg/kg doses resulted in a significant increase in phospho‐Tie2:total Tie2 ratio. (e) Urinary albumin to creatinine ratio (uACR) was measured before treatment initiation (baseline) and after 2 and 4‐weeks of treatment with lisinopril or mAb 109.1. Lisinopril treatment resulted in a significant decrease in uACR after 2 and 4‐weeks of treatment compared to vehicle. mAb 109.1 treatment at both doses resulted in no significant change in uACR after 2 and 4 weeks of treatment. (f) Plasma creatinine (g) Blood Urea Nitrogen (BUN) and (h) Blood glucose was measured in the DKD mice after 4‐weeks of treatment with lisinopril or mAb 109.1. Treatment with lisinopril or mAb 109.1 at both doses exhibited no significant changes in plasma creatinine, BUN and blood glucose compared to vehicle treatment. *n* = 14–16 mice per treatment group. VEPTP, vascular endothelial protein tyrosine phosphatase; DKD, diabetic kidney disease; AAV, adeno‐associated virus; s.c., subcutaneous; Tie2, tyrosine kinase with immunoglobulin and epidermal growth factor homology domains 2.

Four weeks after renin‐AAV injection, mice were randomized based on urinary albumin: creatinine ratio, systolic blood pressure, blood glucose, and body weight. Mice were divided into four treatment groups and treated with either vehicle, lisinopril (10 mg/kg/day) in drinking water or *s.c*. injections of the VEPTP mAb 109.1 at doses of 5 and 20 mg/kg administered twice per week. The age of the mice and corresponding timeline of renin‐AAV injection and drug treatment are shown in Figure 4a. Lisinopril, an ACE inhibitor, was used as a positive control in this study. Lisinopril dose selected was within standard ranges previously reported in mouse models of DKD (Wu et al., 2022). The doses for the VEPTP mAb 109.1 were selected from pharmacokinetics experiments as described above. As shown in Figure 4b, body weight continued to increase with time in all treatment groups, however these increases were not significantly different from baseline. Furthermore, no significant difference in body weight were noted in the mAb 109.1 or lisinopril treated mice compared to vehicle treatment. As shown in Figure 4c, the plasma concentrations of the VEPTP mAb 109.1 at 5 and 20 m/kg were 77 and 161 μg/mL respectively at the end of the 4 weeks of treatment. As shown in Figure 4d, treatment with lisinopril and the VEPTP antibody at both doses resulted in a significant increase in kidney Tie2‐phosphorylation.

As reported previously, urine ACR was significantly reduced with lisinopril treatment at both 2 and 4 weeks compared to vehicle treatment (Figure 4e). However, mAb 109.1 treatment at either dose did not improve uACR. No changes in plasma creatinine, BUN and glucose were noted with lisinopril or mAb 109.1 treatment at either dose compared to vehicle treatment (Figure 4f–h).

### 3.5 Effects of VEPTP inhibition with mAb 109.1 on kidney mRNA expression of endothelial activation, fibrotic, inflammatory, and injury markers in the db/db uninephrectomized ReninAAV mice model for DKD

**FIGURE 5:**
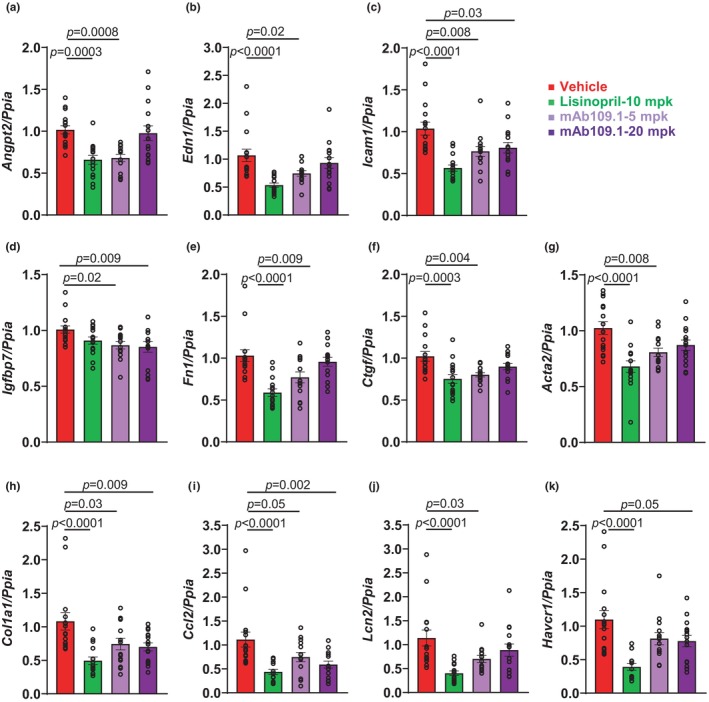
Effects of VEPTP inhibition with mAb 109.1 on kidney mRNA expression of endothelial activation, fibrotic, inflammatory and injury markers in the db/db uninephrectomized ReninAAV mice model for DKD. Female db/db uninephrectomized ReninAAV mice were injected with either vehicle, lisinopril or two doses (5 and 20 mg/kg) of mAb 109.1 for 4‐weeks. After treatment completion kidneys were collected and analyzed for mRNA expression of (a–d) endothelial activation (e–h) fibrosis (i, j) inflammatory and (k) kidney injury marker by qPCR. *n* = 14–16 mice per treatment group. Angpt2, angiopoietin 2; Edn1, endothelin 1; Icam1, intercellular adhesion molecule 1; Igfb7, insulin like growth factor binding protein 7; Fn1, fibronectin 1; Ctgf, connective tissue growth factor; Acta2, actin alpha 2 smooth muscle; Col1a1, collagen type I alpha 1 chain; Ccl2, chemokine (C‐C motif) ligand 2; Lcn2, lipocalin 2; Havcr1, hepatitis A virus cellular receptor 1; Ppia, peptidylprolyl isomerase A.

To determine the effects of VEPTP inhibition with mAb 109.1 on kidney endothelial activation, fibrosis, inflammation, and injury in DKD, we determined the mRNA expression of endothelial activation markers *Angpt2*, *Edn1*, *Icam1* and *Igfbp7*, fibrosis markers *Fn1*, *Ctgf*, *Acta2* and *Col1a1*, inflammatory markers *Ccl2*, *Lcn2*, and kidney injury marker (*Havcr1*) transcripts in the kidneys of DKD mice (db/db Unx ReninAAV) treated with the VEPTP blocking antibody or lisinopril. As shown in Figure 5a,b, VEPTP mAb 109.1 treatment at 5 mg/kg caused a significant decrease in the expression of *Angpt2* (32%) and *Edn1* (25%) compared to vehicle treatment, however no inhibitory effects on *Angpt2* and *Edn1* were observed with mAb 109.1 treatment at 20 mg/kg. As shown in Figure 5c,d, mAb 109.1 treatment at both doses resulted in a significant decrease in *Icam1* (20%–23%) and *Igfbp7* (13%–15%) compared to vehicle treatment.

VEPTP antibody mAb 109.1 treatment at 5 mg/kg dose resulted in a significant decrease in the expression of the fibrosis markers *Fn1*, *Ctgf*, *Acta2* and *Col1a1* (Figure 5e–h). Furthermore, the expression of *Co1a1* was also significantly decreased with mAb 109.1 treatment at the 20 mg/kg dose. As shown in Figure 5i, VEPTP mAb 109.1 treatment at both doses resulted in a significant decrease in the expression of the cytokine *Ccl2* (25%–41%) compared to vehicle treatment. VEPTP mAb 109.1 treatment at 5 mg/kg resulted in a significant decrease in *Lcn2* expression (Figure 5j). As shown in Figure 5k, the expression of the kidney injury marker Kim1 (*Havcr1*) was significantly decreased with mAb 109.1 treatment at the higher dose of the antibody. As shown in Figure 5a–j, lisinopril treatment in DKD mice resulted in a significant decrease in the expression of endothelial activation, fibrosis, inflammatory, and injury markers tested here, except *Igfbp7*.

## 4 DISCUSSION

DKD is a complex multifactorial disease wherein endothelial dysfunction, endothelial cell loss, and capillary rarefaction in glomeruli and tubulo‐interstitium play an important role in disease development and progression (Advani & Gilbert, 2012; Gilbert, 2014; Jourde‐Chiche et al., 2019; Satchell, 2012). Angiopoietin ligands, their cognate receptor Tie2 and VEPTP play an integral role in glomerular vascular development and preserve microvascular integrity during diabetes (Carota et al., 2019; Jeansson et al., 2011; Siddiqi & Advani, 2013). Genetic deletion of the floxed *Veptp* alleles induced postnatally by doxycycline administered via drinking water for 21 days resulted in almost complete loss of the VEPTP protein expression and decreased albuminuria in the Akita‐Renin DKD mice (Carota et al., 2019). Pharmacological approaches targeting VEPTP with ECD domain targeting antibodies have been reported (Bäumer et al., 2006; Winderlich et al., 2009). However, to the best of our knowledge there is no report characterizing VEPTP inhibition with ECD targeting antibodies in a chronic model for DKD. Therefore, in the current study we set forth to investigate the role of VEPTP inhibition with the ECD targeting antibody mAb 109.1 in the db/db uninephrectomized ReninAAV mouse model. This mouse model was selected because it is the most progressive model for type 2 DKD, sharing common molecular pathways with the human disease and translational relevance of pharmacological treatments (Harlan, Heinz‐Taheny, Overstreet, et al., 2018; Harlan, Heinz‐Taheny, Sullivan, et al., 2018; Østergaard et al., 2021). However, one caveat of this model is that it is in female mice and gender specific effects cannot be ruled out. We found that mAb 109.1 treatment induced Tie2 phosphorylation and reduced VEGF‐A stimulated permeability both in vitro and in vivo. Dose dependent plasma antibody exposure and kidney target engagement/pharmacodynamic marker (Tie2 phosphorylation) were achieved with mAb 109.1 treatment, however, mAb 109.1 treatment did not reduce uACR or albuminuria, plasma creatinine, BUN, and blood glucose. Since mAb 109.1 treatment had no effect on renal endpoint observations, histopathology experiments were not performed. Indeed, as has been reported previously, lisinopril treatment resulted in significant improvements in uACR (Harlan, Heinz‐Taheny, Overstreet, et al., 2018; Harlan, Heinz‐Taheny, Sullivan, et al., 2018; Østergaard et al., 2021), but did not decrease plasma creatinine (Harlan, Heinz‐Taheny, Overstreet, et al., 2018), BUN, and blood glucose in this mouse model of DKD. The effects of lisinopril on kidney histology in this mouse model has been characterized previously (Harlan, Heinz‐Taheny, Overstreet, et al., 2018; Østergaard et al., 2021).

Genetic deletion of VEPTP decreased albuminuria in DKD mice (Carota et al., 2019), however pharmacological inhibition with mAb 109.1 in the current study did not reduce albuminuria. Factors that may have led to this difference in observation could be the different genetic background, sex and DKD mouse models used in both studies being different and the fact that the ReninAAV Akita/129 mice exhibit lower uACR than db/db Unx ReninAAV mice (Harlan, Heinz‐Taheny, Sullivan, et al., 2018). Although both models are characterized by histological changes, these changes appeared more pronounced in the db/db Unx ReninAAV mice (Harlan, Heinz‐Taheny, Sullivan, et al., 2018). In this context VEPTP inhibition in a mouse model with less severe phenotype or earlier intervention in the current study may have yielded different outcomes. Another plausible reason for the lack of uACR reduction with mAb 109.1 could be that the “outside‐in” approach of targeting VEPTP ECD is not sufficient to inhibit the intracellular phosphatase activity of the receptor. VEPTP small molecule inhibitors are cell permeable and act directly on the phosphatase domain of the receptor, instead mAb 109.1 is a large cell impermeable molecule targeting the VEPTP ECD. Supporting this notion, it was observed that the VEPTP small molecule inhibitor AKB‐9778 led to a moderate reduction in albuminuria in patients with diabetic macular edema (Kevin et al., 2018). These data would argue that targeting the intracellular phosphatase domain is required to restore endothelial homeostasis.

The VEPTP KO data supports the concept that in the VEPTP knockout mice, in the complete absence of the VEPTP protein, additional downstream signaling mediated by other substrates (Drexler et al., 2019; Vestweber, 2021) are impacted. VEPTP targets include critical endothelial players such as Tie2, VE‐cadherin, VEGFR2 and FGD5 (Drexler et al., 2019; Hayashi et al., 2013; Mellberg et al., 2009; Vestweber, 2021). Indeed, it has been shown that VEPTP and VEGFR2 are found in close proximity (< 40 nm) and silencing VEPTP enhances VEGFR2 tyrosine phosphorylation (Hayashi et al., 2013; Mellberg et al., 2009). Furthermore, recently additional novel substrates of VEPTP have been identified such as PECAM‐1, claudin‐5, EphB4, and cortactin, all of which are known to play an important role in maintaining endothelial homeostasis (Drexler et al., 2019). Although the general notion has been that the vessel stabilizing effects of VEPTP inhibition are mediated mostly by Tie2, these recent observations would argue that VEPTP functions as an important master regulator of several critical endothelial targets and thereby regulates overall vascular homeostasis.

Targeting VEPTP with mAb 109.1 interferes with VEPTP and binding partner interactions limited to the VEPTP ECD, whereas in complete absence of the protein vascular homeostasis mediated by a plethora of VEPTP substrates will be impacted. Furthermore, it is possible that a yet unidentified VEPTP ligand could induce uptake of VEPTP and thereby indirectly enhance Ang‐1 driven activation of Tie2 (Vestweber, 2021). Limitations of the current study are that although we have studied the effect of VEPTP inhibition with mAb 109.1 on Tie2 phosphorylation, we have not explored the effects of mAb 109.1 on other critical endothelial substrates such as VEGFR2 and VE‐cadherin, the renal endpoints observations with the VEPTP antibody mAb 109.1 treatment were in female db/db uninephrectomized ReninAAV DKD mice and the current study lacks histopathology analyses.

In summary, here we show that VEPTP inhibition with an ECD targeting mAb 109.1 induces Tie2 phosphorylation and improves VEGF‐A induced vascular permeability both in vitro and in vivo. Despite dose dependent increase in plasma antibody exposure and kidney target engagement, mAb 109.1 treatment did not result in renal function improvement in the db/db Unx ReninAAV mouse.

## AUTHOR CONTRIBUTIONS

**M. C. M.** conceived the study. **R. R.**, **T. A. N.**, **P. K.**, and **P. P.** designed research. **R. R.**, **T. A. N.**,**P. K.**, **A. B. M.**, **V. C.**, **K. P. F.**, **W. Z.**, **G. Z.**, and **N. O. B.** performed experiments, analyzed data, and interpreted results of experiments. **R. R.** prepared figures, and drafted manuscript. **R. R.**, **T. A. N.**, **A. P.**, and **M. C. M.**, edited and revised manuscript.

## CONFLICT OF INTEREST STATEMENT

All authors are either current or previous employees of Johnson & Johnson Research & Development, which provided all funding for the research.

## ETHICS STATEMENT

All animal experiments were performed in accordance with the Institutional Animal Care and Use Committee (IACUC) and Charles River Accelerator and Development Lab (CRADL) guidelines.

## Data Availability

Data will be made available upon reasonable request.
